# One-Step Lower Leg Reconstruction with Vascularized Functional Vastus Lateralis Muscle Flap in the Treatment of Embryonal Rhabdomyosarcoma for a Six-Month-Old Boy: A Case Report

**DOI:** 10.3390/medicina56070362

**Published:** 2020-07-21

**Authors:** Dzintars Ozols, Marisa Maija Berezovska, Janis Krasts, Marika Grutupa, Aigars Petersons

**Affiliations:** 1 Department of Pediatric Surgery, Riga Stradins University, Lv-1007 Riga, Latvia; marisamaija@gmail.com (M.M.B.); aigars.petersons@rsu.lv (A.P.); 2 Department of Pediatric Surgery, Childrens Clinical Univercity Hospital, Lv-1004 Riga, Latvia; janis.krasts@bkus.lv; 3 Department of Hematooncology, Childrens Clinical Univercity Hospital, Lv-1004 Riga, Latvia; marika.grutupa@bkus.lv

**Keywords:** embryonal rhabdomyosarcoma, functional vastus transplantation, infant microsurgery

## Abstract

Rhabdomyosarcoma (RMS) is a common soft tissue sarcoma in childhood, however, it is very rare in the neonatal period (0.4–2% of cases). This case depicts a boy, who presented with RMS at two weeks of age, but officially diagnosed at the age of three months. MRI and scintigraphy determined a soft tissue tumor in the soleus muscle, while biopsy confirmed embryonal RMS with high mitotic activity (Ki67 (monoclonal antibodies) ~80%). CWS (Cooperative Weichteilsarkom Studiengruppe)-2012 with I2VA (ifosfamide, vincristine, actinomycin) chemotherapy regimen was administered per protocol. Surgical treatment was performed at age of six months and 18 days. The operation consisted of radical tumor resection and total triceps surae with partial fibula resection. Immediate reconstruction of triceps muscle was accomplished using a vascularized functional musculocutaneous vastus lateralis flap. Functional outcome was measured using the Lower Extremity Functional Scale (LEFS) and the Foot and Ankle Outcome Score (FAOS) with the results of 92.5% and 99% respectively.

## 1. Introduction

Pediatric soft tissue sarcomas are part of a heterogeneous group of tumors originating from embryonic mesodermal tissues during the process of differentiation into various mesenchymal tissue components of the human body. These tumors constitute 6% to 8% of all cancers in children less than 15 years of age [1], however, it is very rare in the neonatal period as its incidence is 0.4–2% of cases with male predisposition [2]. Surgery remains the standard of treatment, yet the curative aim cannot be achieved without adjuvant treatment. Pre-treatment staging and risk classification are of prime importance in selecting an effective treatment protocol. Tumor resectability, the response to induction chemotherapy and radiation generally determine the risk group, and these factors are functions of tumor site, size, and biology. Surgery provides the best choice for local control of small resectable tumors in a favorable site. Reconstructive possibilities are limited in the pediatric population younger than two years of age due to relatively small structures such as blood vessels [3]. There are paucity of articles showing microvascular reconstruction cases for patients at the age of four to six months. Well-known microvascular flaps such as the latissimus dorsi (LD) or rectus abdominis muscle flap have been reported to close defects for pediatric patients at an infant age [4,5,6]. The operation is challenging and needs to be led by experienced surgeons due to the small size of vessels and risk of vasospasms. 

## 2. Case Report

A three-month-old boy is admitted for investigations as his left lower leg is much larger than his right lower leg. On admission, the circumference, which measured at 4–5 centimeters (cm) below the patella at the thickest part of the lower leg on the left side, was 23 cm while the right side was 18.5 cm. This mass was palpable at the age of two weeks and had exponential growth. He is the first child and pregnancy to term, and was born at 41 weeks. However, during delivery he experienced asphyxia and aspiration with an Apgar score of 3/4/6. He had a normal birthweight of 4200 g and neonatal investigations of neurosonography, abdominal ultrasonography (USG), and echocardiogram determined no pathologies. His mother worked in carpentry, where she had contact with chemical compounds while pregnant. 

**Figure 1:**
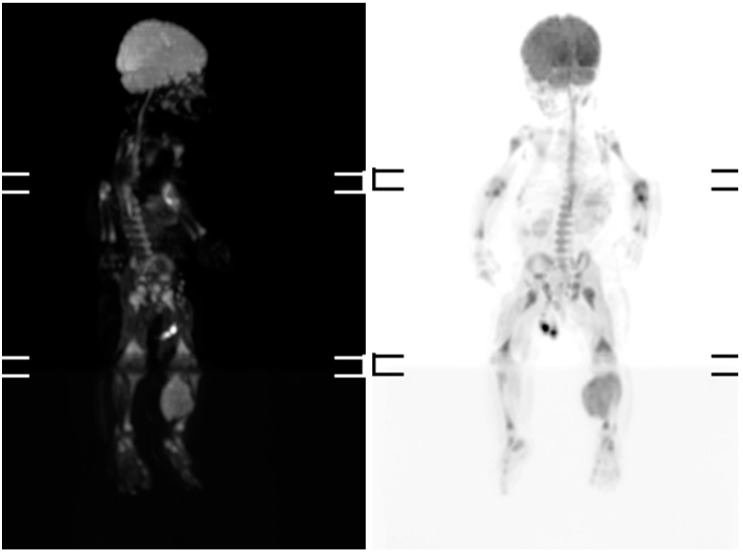
Scintigraphy at three months old, revealing a soft tissue mass in the left calf.

**Figure 2:**
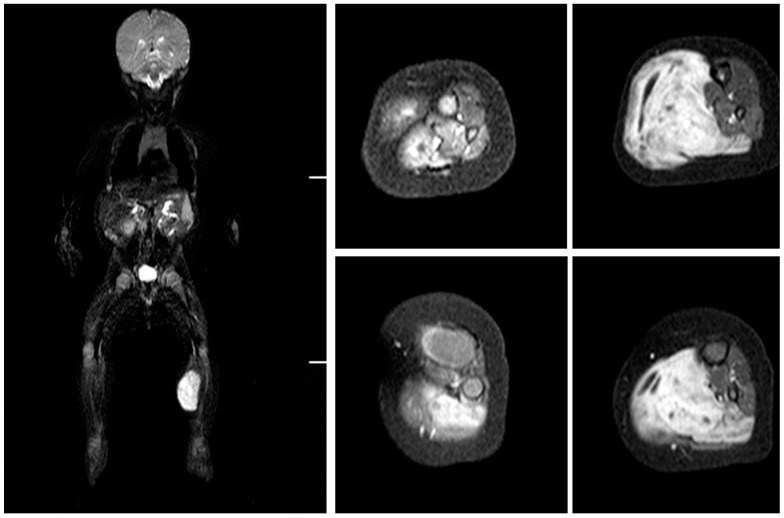
Magnetic resonance imaging (MRI) at three months old. RMS (rhabdomyosarcoma) mass in the superficial posterior compartment of the left lower limb.

Magnetic resonance imaging (MRI) and scintigraphy determined a soft tissue tumor in the soleus muscle, while biopsy confirmed embryonal rhabdomyosarcoma with high mitotic activity (Ki67 (monoclonal antibodies) ~80%) (Figure 1 and Figure 2). 

CWS (Cooperative Weichteilsarkom Studiengruppe)-2012 with I2VA (ifosfamide, vincristine, actinomycin) chemotherapy regimen was administered per protocol after a port-a-cath was inserted. The treatment comprises 9 courses I2VA with Ifosfamid (as three-hour infusion daily on Days 1 and 2 of each course), Vincristine (as single intravenous bolus injection on Day 1 of each course with two additional injection on Day 1 of Week 2, 3, 5, 6) and Actinomycin-D (as single intravenous bolus injection on Day 1 of each course) with prophylactic support of 24 h hydration with uromitexan, intravenous (i/v) dezintoxication, ondansetron, methylprednisolone, and per os (p/o) biseptol. Interval between the courses is three weeks. During chemotherapy, he needed erythrocyte and thrombocyte transfusions. He received four rounds of chemotherapy before undergoing radical surgical treatment at the age of six months and 18 days (202 days old). On the operation day, his left lower leg circumference was the same as that of the right lower leg—18 cm.

Operation started with a longitudinal incision of the lower leg to secure a wide opening and good visualization for the radical tumor resection. The open biopsy skin scar, total superficial posterior compartment and proximal section of fibula were resected to obtain radical tumor excision with at least two centimeters margins. The gastrocnemius, plantaris and soleus muscles were resected at the proximal level two centimeters from their origin and distally at the Achilles tendon level two centimeters from their insertion in calcaneus. No functional muscles were left in posterior superficial compartment. Deep posterior compartment muscles and structures were left intact as the tumor was localized at the lateral part of the triceps surae muscle. Longitudinal osteotomy of fibula with fibular proximal growth plate was performed for the radical removal of the lateral insertion of soleus muscle. Lateral compartment muscles and structures were preserved. The tibial nerve and posterior tibial artery were preserved, while two gastrocnemius motor branches were dissected for functional muscle reinnervation.

Immediate reconstruction of posterior muscle group was accomplished using a vascularized musculocutaneous vastus lateralis flap. The ipsilateral leg was used to minimize donor site morbidity as amputation is the next step if there is tumor recurrence. A ten-centimeter incision was rendered in the lateral side of femur, the fascia latae was separated and therefore, the quadriceps muscles exposed. Vastus lateralis muscle was dissected at the proximal level near to great trochanter and distally at the tendon. Two centimeters of the quadriceps tendon was included in flap for the Achilles tendon reconstruction. ALT (anterior lateral thigh) perforator with skin paddle was also included in composite flap. The separated fascia latae was not included in flap.

**Figure 3:**
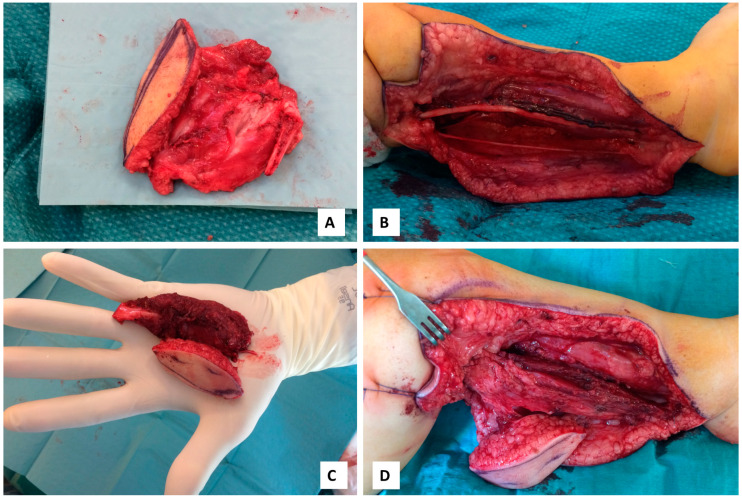
The tumor successfully resected with the tumor’s approximate size of 5 × 6 × 2 cm (**A**). Lower leg after radical tumor removal with a preserved tibial nerve (**B**). Functional vastus lateralis myocutaneous flap with ALT (anterolateral thigh) perforator based skin paddle (**C**). Microvascular flap with vascular supply after anastomoses (**D**).

ALT perforator was rising from the vascular branch of vastus lateralis muscle and directing through the muscle. Lateral femoral circumflex artery with two concomitant veins were dissected to established blood supply to vastus lateralis muscle flap. Artery size was measured at 0.4–0.6 mm at the level of dissection. Two motor branches of the vastus lateralis muscle were visualized and included in flap for motor reinnervation. Total size of vastus muscle 8 × 4 cm with 4 × 2 cm skin perforator flap. End-to-end posterior tibial artery and two concomitant veins anastomoses were realized with 10/0 nylon sutures. Two gastrocnemius muscular nerves were used to secure reinnervation for the vastus lateralis muscle flap. Vastus lateralis muscle was attached to the gastrocnemius muscle’s origin using absorbable sutures and distally was sutured to Achilles tendon using absorbable sutures (Figure 3). Donor site was realized with primary closure. The operation was completed in six hours and 40 min. No anticoagulants were used in postoperative period. Simple rehydration using Ringer lactate was used with targeting the hematocrit level less than 30%. 

The histology report confirms the immunohistochemical profile of embryonal rhabdomyosarcoma, however, separate tumor cells constituted on one of the resected edges. Therefore, the tumor was removed within neoplastic tissue borders, but it did not change the chemotherapy or require additional radiation therapy. The patient continued postoperative chemotherapy with his fifth round requiring filgrastim due to neutropenia and continual antifungal prophylaxis (fluconazole, clotrimazole). After the seventh round of chemotherapy, a control MRI determined possible residual tumor tissues 2.4 × 0.8 × 2.2 cm with unclear borders that absorbs contrast. USG controlled puncture biopsy was performed and the histology determined cutaneous and subcutaneous soft tissue with fibrosis and non-specific chronic granulomatous inflammatory inflammation, however, there is no definite data about malignancy in this material. Removed tissues were interpret as postoperative scaring tissues associate with postoperative hematoma. In total, the patient received nine rounds of chemotherapy. He completed treatment with port removal at the age of eight months and 23 days (327 days old). 

**Figure 4:**
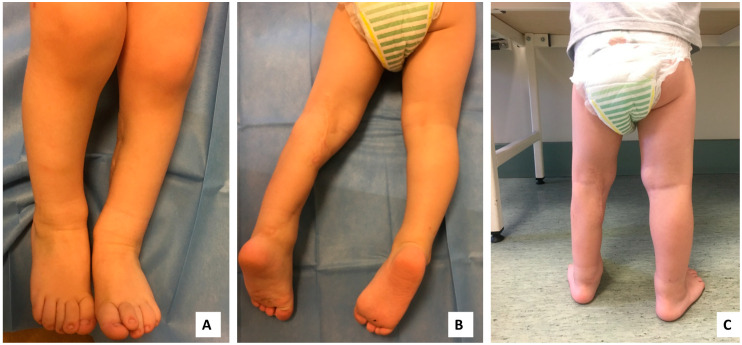
Two-year follow-up, patient demonstrated no physical limitations (**A–C**).

**Figure 5:**
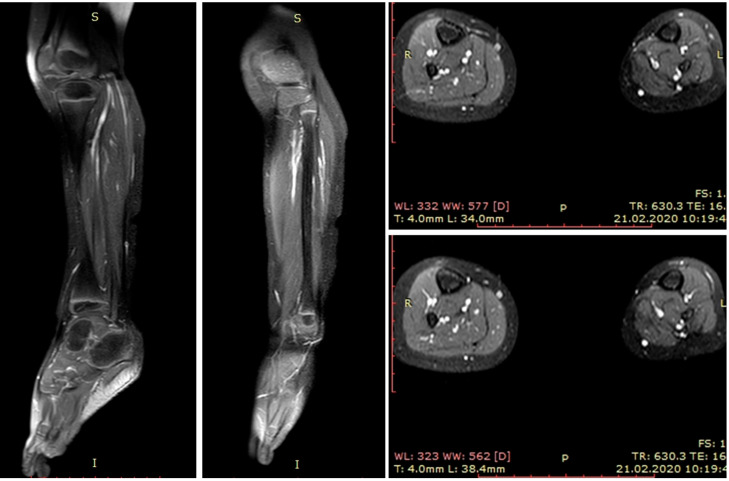
Magnetic resonance imaging (MRI) at the two years follow up. Reconstructed superficial compartment with functional vastus muscle flap can be visualized and no RMS (rhabdomyosarcoma) recurrence present. The healthy right size approximately 30% bigger comparing reconstructed triceps muscles.

During the postoperative period, no tumor recurrence or metastases were observed. At the two-year follow-up, the patient’s left side was visibly smaller than the right, however, he performed all lower leg movements without hindrances (Figure 4). The Medical Research Council’s scale (MRC scale) was used to evaluate muscle power of the limb. The MRC scale of posterior compartment muscle strength for the movement of the talocrural joint scored 4–5 points as strength was little bit lower in comparison to the healthy side. The MRC for quadriceps muscle for the movement of knee joint scored 5 points as there were no differences with healthy side. Lateral compartment muscles MRC for movement of talocrural joint scored 5 points and deep posterior compartment muscles MRC scored 5 points for toe flexion. Sensation innervation at the peroneal and tibial nerve zones of the foot were intact and the patient reacted to the pain using a two-point discriminatory test. Decreased sensation was found at the lateral part of the foot and lateral malleolar side. Range of motion (ROM) of the knee joint flexion 0–140° (reconstructed) and contralateral 0–145°, ankle joint plantar flexion 0–40° and dorsiflexion 0–20° (reconstructed), and the contralateral ankle joint plantar flexion 0–45° and dorsiflexion 0–20°. LEFS (Lower Extremity Functional Scale) was 92.5% as he still is not allowed to enter the bath by himself and FAOS (Foot and Ankle Outcome Score) was 99%. The patient’s physical development is on track with other children his age (Supplementary Video S1). There is a great prognosis as control diagnostics have not shown any new tumors or distant masses (Figure 5). 

### Ethics Approval and Consent to Participate Investigations Were Approved by the Institutional Ethical Review Board

The study protocol was approved on 28.02.2019. by the Ethics and Scientific committee of the Riga Stradins University Ethics committee Nr.6-3/2/47 and ICF (informed consent form) was signed by his parents before treatment.

## 3. Discussion

Embryonal RMS is rare tumor with good prognostic outcome [7]. Treatment recommendations for limb RMS combine chemotherapy and surgical tumor removal with secondary functional reconstruction in comparison with previous recommendations consisting of radical excision, primary or secondary amputation combined with irradiation therapy [8]. Currently, modern treatment of malignant RMS tumors requires combined treatment: chemotherapy and radical excision with primary or secondary reconstruction to restore functionality and improve quality of life [9,10]. Functional muscle transplantation for oncologic patients are widely used to restore functionality of the limb in adults, but there is paucity of articles for functional muscle transplantation at the infant age as vessel size are extremely small [11,12,13]. 

We demonstrate a complex treatment of the embryonal RMS with one-step surgical reconstruction with functional vastus lateralis myocutaneous microvascular flap for patient at the age of six months. Small vessel size is challenging for microvascular anastomosis at the infant age, but refined surgical skills and excellent magnification can lead to successful surgical outcomes. LD flap is the first choice for the reconstruction of muscle defects due to well-known vascular anatomy, but for tumor reconstruction cases, there is always a risk for recurrence and late limb amputation. Therefore, treatment within same anatomical region (ipsilateral limb) minimizes the possible donor site morbidity as there is no necessity for a functional quadriceps muscle post transfemoral amputation; however, there would be a stronger requirement for upper extremity muscles to use crutches and to obtain better balancing when using a prosthesis.

Functional reconstruction of triceps surae allowed the patient to start walking at age of 15 months without noticing any differences. The two-year follow-up results showed good functional outcomes and no signs of tumor recurrence. Maternal concern lead to prompt tumor identification, which influenced successful treatment without radical amputation of the left lower leg. 

Regular follow-up needs to occur to reduce risk of tumor recurrence for the embryonal RMS patients using MRI or positron emission tomography (PET) scan, but to bear in mind that microvascular flap reconstruction scar tissues or postoperative seroma can be misdiagnosed as a malignant process. Experienced radiologists can clarify the diagnosis or surgical biopsy with histological evaluation, which must be performed to minimize the risk of tumor recurrence.

## 4. Conclusions

Embryonal rhabdomyosarcoma is a rare congenital tumor with a good prognostic outcome. We provide a successful treatment with maximal functional restoration using microvascular tissue transplantation techniques in the infant period. We recommend the radical resection of the tumor and functional reconstruction using microvascular flaps for pediatric patients younger than one year of age. 

## Supplementary Materials

The following are available online at https://www.mdpi.com/1010-660X/56/7/362/s1, Video S1: Lower leg functionality at the two-year follow-up visit.
